# Open questions and research gaps for monitoring and updating AI-enabled tools in clinical settings

**DOI:** 10.3389/fdgth.2022.958284

**Published:** 2022-09-02

**Authors:** Sharon E. Davis, Colin G. Walsh, Michael E. Matheny

**Affiliations:** ^1^ Department of Biomedical Informatics, Vanderbilt University Medical Center, Nashville, TN, United States; ^2^ Department of Medicine, Vanderbilt University Medical Center, Nashville, TN, United States; ^3^ Department of Psychiatry, Vanderbilt University Medical Center, Nashville, TN, United States; ^4^ Department of Biostatistics, Vanderbilt University Medical Center, Nashville, TN, United States; ^5^ Tennessee Valley Healthcare System VA Medical Center, Veterans Health Administration, Nashville, TN, United States

**Keywords:** dataset shift, model updating, machine learning, risk model surveillance, artificial intelligence

## Abstract

As the implementation of artificial intelligence (AI)-enabled tools is realized across diverse clinical environments, there is a growing understanding of the need for ongoing monitoring and updating of prediction models. Dataset shift—temporal changes in clinical practice, patient populations, and information systems—is now well-documented as a source of deteriorating model accuracy and a challenge to the sustainability of AI-enabled tools in clinical care. While best practices are well-established for training and validating new models, there has been limited work developing best practices for prospective validation and model maintenance. In this paper, we highlight the need for updating clinical prediction models and discuss open questions regarding this critical aspect of the AI modeling lifecycle in three focus areas: model maintenance policies, performance monitoring perspectives, and model updating strategies. With the increasing adoption of AI-enabled tools, the need for such best practices must be addressed and incorporated into new and existing implementations. This commentary aims to encourage conversation and motivate additional research across clinical and data science stakeholders.

## Introduction

As the implementation of artificial intelligence (AI)-enabled tools is realized across diverse clinical environments, there is a growing understanding of the need for ongoing monitoring and updating of prediction models (1–5). Beyond initial validation and local tailoring of models transported across settings, temporal deterioration in model accuracy after development has been documented across clinical domains and settings (6–10). Neither regression nor advanced machine learning algorithms are exempt from these temporal changes in performance (8, 11). Such performance drift degrades the clinical utility of AI-enabled tools, jeopardizes user trust, and poses safety concerns when insufficiently accurate predictions are used in decision-making (1, 6, 12, 13).

Dataset shift (14)—temporal changes in clinical practice, patient populations, and information systems—is well-documented as a source of performance drift and recognized as a challenge to the sustainability of AI-enabled tools in clinical care (6–8, 15–17). Model developers and system managers have access to a variety of approaches to address performance drift and underlying dataset shift in order to restore model performance to clinically acceptable levels. In some cases, model performance may be restored by correcting technical errors introduced by structural changes in information systems, such as implementation of revised data standards. However, in many cases where dataset shift is more nuanced and multifaceted, model updating through recalibration, retraining, or revision will be required. While best practices are well-established for training and validating new AI models (18), there is limited guidance on prospective validation and few best practices for model monitoring and updating.

In this paper, we highlight the need for maintaining clinical prediction models and discuss open questions regarding this critical aspect of the AI modeling lifecycle. First, we illustrate performance drift across models implemented in the production electronic health record (EHR) system at an academic medical center. Second, we discuss several open research questions and describe the nuances required for best practice guidance. Despite advances in continuous learning algorithms that evolve models as data accrue, such algorithms are subject to additional challenges and healthcare applications still predominantly rely on static models that will require periodic updating (19). Although we focus our discussion on updating static models, similar questions may arise around surveillance practices for continuous learning models.

## Performance drift in operational models

Most studies documenting temporal model performance have been conducted in registry or research datasets rather than with operational data from models running in real-time clinical settings (7–9, 16). However, the transition from a retrospective research frame to real-time operational implementation may impact performance as input mappings change and the timing data availability shifts (20–22). To explore performance drift in an operational setting, we evaluated the performance of two models currently implemented in the production EHR system at Vanderbilt University Medical Center (VUMC): a non-proprietary, externally developed model predicting readmission (LACE+) (23) and a locally developed model predicting suicidal behaviors (Vanderbilt Suicide Attempt and Ideation Likelihood model, VSAIL) (24).

Table 1 provides an overview of each model, highlighting differences in modeling methods, training cohorts, and intended use. We extracted stored predictions calculated in real-time and outcomes associated with each prediction using data available in VUMC's EHR. For the LACE+ model, we note that this approach may undercount readmissions if patients were readmitted to a different medical facility. Monthly performance was evaluated using metrics relevant to each model's intended use. We measured the mean calibration of the LACE+ readmission model with the observed to expected outcome ratio (O:E) and clinical utility of the VSAIL suicidality model with the number needed to screen (NNS; the inverse of positive predictive value).

**Table 1:** Prediction models evaluated for temporal validation of real-time scores generated within a production electronic medical record system.

Details	LACE+	VSAIL
Outcome	30-day readmission	30-day suicidal ideation or attempt
Intended use	Quality benchmarking using predicted risk of readmission calculated at discharge	Clinical decision support delivered at arrival for inpatient and outpatient encounters
Development setting	Patients from multiple hospitals in Ontario, Canada	VUMC patient population
Modeling approach	Logistic regression	Random forest
Evaluation period	January 2018 through March 2022	December 2019 through January 2022

VUMC, Vanderbilt University Medical Center.

**Figure 1:**
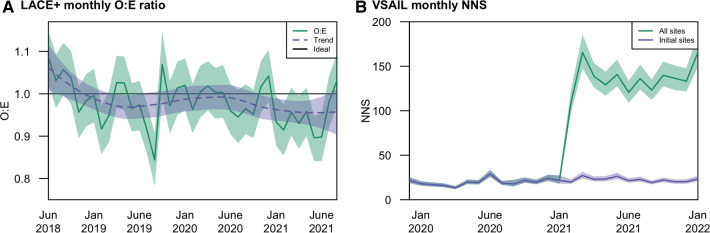
Temporal performance at Vanderbilt University Medical Center of the (**A**) LACE+ readmission model in terms of mean calibration (O:E); and (**B**) VSAIL suicidality model in terms of number needed to screen (NNS).

The LACE+ model, locally calibrated to the VUMC population, sustained performance over the evaluation period (Figure 1A). Monitoring highlighted the importance of distinguishing noise from both informative local change in performance and true model deterioration. Over the first 2.5 years, variability in observed O:Es did not follow a significant trend. In the last year of evaluation, however, there may be a trend toward lower O:Es. Depending on the use case, this declining O:E could be seen as indicating improved local quality (i.e., reducing readmissions) or increasing miscalibration. We note that O:E, a crude measure of calibration, may conceal calibration drift within clinically important risk ranges (25). VSAIL maintained a relatively stable NNS during the first year of implementation (median monthly NNS = 19), with the NNS abruptly increasing in February 2021 (median monthly NNS = 136); Figure 1B). This shift corresponds to operational changes in implementation, with the model being applied to a much broader patient population. Within the original population, VSAIL's NNS remained stable (median monthly NNS = 22). The higher NNS in the broader population may still be feasible but should be considered in the implementation team's cost-benefit analysis and may warrant further investigation of performance in select clinical settings or subpopulations. These findings illustrate performance drift in a single health system's EHR and contribute to the mounting evidence that AI-enabled tools require long-term strategies to understand performance trajectories and maintain utility.

## Research and best practice gaps in model maintenance

Despite concerns over the long-term stability of model performance, health systems lack generalizable guidance for operationalizing post-implementation maintenance strategies. To develop guidance and establish best practices, additional research and debate are needed in three focus areas: model maintenance policies, performance monitoring perspectives, and model updating strategies (Table 2).

**Table 2:** Overview of gaps in best practices for model maintenance.

Domain	Gaps/Needs
Maintenance policies	
How should model ownership impact local control over maintenance?	• Policies establishing updating expectations of proprietary models • Clarity and fairness of local updating opportunities of proprietary models • Prototypes for establishing collaborative updating of multi-system owned models
How do we ensure comparable performance across demographic groups is sustained during the maintenance phase?	• Guidance on whether and when changes in model fairness warrant pausing AI-enabled tools • Methods for addressing performance fairness drift when model performance deteriorates differentially across subpopulations
How do we communicate model changes to end users and promote acceptance?	• Design of effective communication strategies for warning end users of model performance drift and informing users when updated models are implemented • Guidance on aligning messaging with end-user AI literacy
Performance monitoring	
At what level should model performance be monitored and maintained?	• Guidance on aligning monitoring and maintenance with use case needs • Recommendations for handling monitoring in smaller health systems, including determining minimum sample size and methods for collaborative monitoring • Policies supporting collaborative model maintenance in low data resource settings • Guidance on managing interim periods of local performance drift between releases of proprietary models that cannot be locally updated
What aspects of performance should be monitored?	• Generalization recommendations on frequency and sample sizes for measuring performance across a variety of metrics • Customizable and expandable tools to monitor a matrix of metrics • Guidelines for aligning metrics of interest with use case needs
How do we define meaningful changes in performance?	• Framework for selecting drift detection methods • Guidance on establishing clinically acceptable ranges of performance and defining clinically relevant decision boundaries • Methods for tailoring drift detection algorithms to detect a clinically important change
Are there other aspects of AI models that we should monitor, in addition to performance?	• Approaches to systematically surveil external features that may impact model inputs and for monitoring input data distributions • Guidance on when to update in response to changes in model inputs if performance remains stable • Systems for disseminating information on changes anticipated to affect common AI models
Model updating	
What updating approaches should be considered?	• Approaches to optimizing update method selection based on performance characteristics most relevant to use case needs • Expanded suite of testing procedures options for more updating methods and increased computational efficiency • Guidance on defining acceptable performance and methods to determine which updating methods, if any, restore acceptable performance
Should clinically meaningful or statistically significant changes in performance guide updating practice?	• Guidance on whether to update models when statistically significant improvement is possible but updating would not provide a clinically meaningful improvement • Methods for comparing updating options that incorporate tests for both statistical and clinical significance • Recommendations for decision-making in cases where available updating methods do not restore performance to acceptable levels
How do we handle biased outcome feedback after model implementation?	• Recommendations for assessing feedback from effective AI-enabled interventions • Methods for model development, validation, and updating that are robust to confounding by intervention

### Maintenance policies

Oversight policies at the health system level could facilitate the maintenance of a portfolio of models by defining a consistent, systematic groundwork for sustaining both new and existing AI-enabled tools. System-level policies can also inform use case parameters to consider when establishing model-specific maintenance plans.

#### How should model ownership impact local control over maintenance?

Health systems certainly have a right and duty to monitor the local performance of the AI models they implement, regardless of where those models originated. However, how to address deteriorations in performance is complicated by model ownership and licensing restrictions. For models developed in-house and local implementations of models in the public domain, health systems have full control over maintenance approaches and may consider the full spectrum of updating methods. At VUMC, the local VSAIL model will be retrained using more recent data and subsequently maintained through a tailored data-driven surveillance approach. When models are developed in collaboration across health systems, best practices could guide collaborative updating and establishment of parameters for local model adjustments.

Updating proprietary models is particularly challenging, despite locally documented drift having required the deactivation of proprietary AI-enabled clinical tools (6). Licenses may restrict updating by not permitting local model recalibration or retraining. Updating options may be further limited by inadequate documentation of training methods (26). Proactive updating of proprietary models by model owners, such as semi-annual updates of the National Surgical Quality Improvement Program (ACS NSQIP) risk models, may alleviate some, but not all, of the need for local updating options. Health systems, national organizations, and policymakers should advocate for more complete documentation of proprietary models and increased access to updating options. This may include the relaxation of local updating restrictions; clear documentation of owner-driven maintenance plans; and proactive, transparent dissemination of updated models to all customers. Enabling such expectations of model owners will require more detailed and consistent guidance on model updating practices covering the concerns described throughout this paper.

#### How do we ensure comparable performance across demographic groups is sustained during the maintenance phase?

Model fairness is now recognized as a critical element of clinical AI models (27, 28). While model fairness comprises a broad set of concerns regarding implementation practices, user uptake and application, and sociotechnical contexts of use (29), fairness also requires models to perform similarly across demographic groups. Establishing initial comparable model performance across subpopulations and subsequently maintaining comparable performance within these groups is thus critical to ensuring model fairness. Novel metrics for evaluating algorithmic fairness across subpopulations are providing insight during model validation and selection of models for implementation in clinical tools (30, 31). A clear next step is to incorporate these new metrics and performance within subpopulations into model monitoring to evaluate fairness over time. This poses new questions regarding how to handle the potential for fairness drift, defined as differential performance drift across subpopulations. Researchers and policymakers will need to address tests for temporal changes in fairness metrics; methods for updating models experiencing fairness drift that prioritize equitable utility for all patients; and whether and when changes in model fairness warrant pausing AI-enabled tools to avoid creating or exacerbating disparities.

#### How do we communicate model changes to end users and promote acceptance?

Open communication between modeling teams and clinical end users is essential to the monitoring and maintenance phase of the AI lifecycle. End users may identify failing AI-enabled tools before performance monitors detect changes in accuracy. They may also provide insight when models are no longer useful from a clinical perspective even with sustained performance, allowing tools to be de-implemented or revised as needed. At the same time, modeling teams should establish policies for disseminating information about model updates to end-users, whether updating is driven by end-user concerns, local model maintenance efforts, or new releases of proprietary models. Such communication, while particularly important for reestablishing trust in models updated in response to end-user concerns, is relevant for all updates. Model maintenance programs need to include specific strategies for this bidirectional communication. Such engagement and transparency regarding model maintenance may also increase acceptance of AI more broadly by assuring users that models are actively being curated, monitored, and assessed with an eye to promoting utility and safety.

The appropriate mode of communication and level of detail provided about model updates are likely to use case-dependent. The ACS NSQIP surgical risk calculator, for example, displays a banner message highlighting recent updates, setting expectations for any noticeable changes in predictions, and eliciting feedback if concerns arise (32). Extensive model revisions or reimplementation of a paused model with restored performance may require more explanation than a banner message can effectively convey. Workflow and communication experts will be key collaborators in designing best practices for disseminating information on model updates. These best practices will likely need to evolve as the health care workforce becomes better trained in AI.

### Performance monitoring perspectives

Ongoing monitoring provides necessary insight into model stability and can alert model managers to concerning performance trends in need of intervention (3, 33, 34). However, insights from monitoring require careful determinations of how model performance is defined and evaluated.

#### At what level should model performance be maintained?

AI models, even when operationalized to meet the needs of a specific health system, may need to be monitored and updated locally, regionally, or nationally. Key features to consider in determining the appropriate level of model maintenance include use case goals, model ownership, and data and analysis resources.

Our understanding of best practices is well-defined in terms of use case and the level of model maintenance. For benchmarking models in quality evaluations, maintenance should be centralized at the largest relevant scope. Stabilizing the performance of quality-oriented models at higher levels imbues local performance deviations with information about variations in care and allows facilities to validly interpret performance trends as indicating improvement or deterioration of local performance over time. For AI-enabled tools aimed at clinical decision-making and population management, individual predictions should be well-calibrated to ensure utility and benefit to patients (13). As a result, more localized monitoring and maintenance are appropriate.

Unfortunately, practical considerations may require centralized monitoring and updating at regional or national scales even when local performance would typically be prioritized. Ownership and licensing requirements of proprietary models may preclude updating models to optimize local performance. Guidance on how to assess and handle local drift in light of such restrictions is necessary to trigger pauses in model implementations when local monitoring efforts reveal concerning performance drift; facilitate communication with end users about paused models and support end users’ information needs during such pauses, and promote timely reporting of issues to model owners.

When local updating is permissible, monitoring and updating remain a challenge for small organizations where data volumes and analytic resources may be limited. Insufficient sample sizes can lead to highly variable performance during monitoring and limit the ability to distinguish performance drift from noise. Smaller organizations, as well as their larger peers, should leverage recent studies by Riley et al. to assess whether sufficient sample sizes are available to validate binary (35), time-to-event (36), and continuous models (35). Recalibration, retraining, and model revision also require sufficient sample sizes (37) and dedicated data science teams that may not be feasible for all organizations. One solution would be to explore whether health information exchanges could be leveraged for collaborative monitoring and updating where local resources are insufficient. Broader research and policy discussions are needed as we think creatively about such multi-level, coordinated efforts to ensure the benefits of predictive tools are available and practical for health care organizations serving all communities.

#### What aspects of performance should be monitored?

While some metrics appear more robust to dataset shift, performance drift has been documented in measures of discrimination, calibration, and clinical utility (7, 8, 10, 16, 38). Monitoring metrics relevant to an AI-enabled tool’s use case is critical to understanding whether changes in performance warrant updating or whether updating may have little impact on model use and outcomes. For example, the number needed to screen was identified by the VSAIL team as the target metric for monitoring and stabilizing model performance as this impacts the cost-benefit analysis of clinics adopting the tool (39). For models deployed in diverse clinical contexts or across multiple tools, tracking a matrix of performance measures would provide insights supporting a variety of user perspectives (12, 40). Monitoring recommendations should thus include components that are agnostic to the performance metrics under consideration (e.g., selection of measurement), as well as components regarding metric selection.

#### How do we define meaningful changes in performance?

Monitoring performance alone is insufficient; model managers need to be able to determine when observed deterioration in performance warrants intervention. Drift detection methods surveil temporal performance to alert users to statistically significant changes (41, 42) and have been applied to monitoring clinical prediction models. (34, 38) Methods vary in their ability to handle multiple forms and speeds of performance drift, as well as in their applicability to clinical contexts where calibration is of interest (43). Best practice recommendations will need to provide a decision framework for selecting between drift detection approaches, including considerations of whether detection algorithms are model-independent; can handle data streams of individual or batched observations, and are flexible in their ability to monitor prediction errors using a variety of metrics.

We note small differences in performance may be detected by the statistical tests underlying drift detection algorithms. However, statistically significant differences in performance may not directly translate into clinically meaningful differences. In such cases, users may question the value of updating or pausing a model in response to detections of small statistically significant, but not clinically important performance drift. The magnitude of acceptable inaccuracy and performance variability likely varies by use case. For example, performance drift is most likely to impact clinical utility when the calibration of predictions near clinically relevant decision thresholds or near classification cut-points deteriorates. Understanding whether, when, and how performance drift affects the clinical utility of predictions for decision-making is key to detecting meaningful changes in monitored models. Defining and measuring clinically acceptable performance and defining clinically relevant decision boundaries remains an open area of research. Subsequent research and guidance will need to address tailoring drift detection algorithms to place more import on clinically important changes in model performance.

#### Are there other aspects of AI models that we should monitor?

In addition to performance metrics, the inputs of AI models could be monitored. This may involve evaluating data streams for changes in predictor distributions and associations (17), as well as establishing teams to actively evaluate external influences in clinical guidelines, software systems, data standards, and health care policies (6). Tracking external influences would allow teams to recognize structural changes that could render a model unreliable and plan customized updating approaches. Changes in data stream features, however, may not necessitate updating unless and until they affect the model accuracy in clinically meaningful ways. Best practices will need to address integrating insights from performance monitoring and evaluations of factors impacting model inputs to promote stable performance while efficiently and conservatively updating models. Additional research could investigate strategies for monitoring these non-performance aspects of AI models and policies for disseminating information across health systems when new practices are anticipated to impact widely adopted models.

### Updating strategies

When updating is initiated by pre-established schedules or detected performance drift, model managers must choose between a range of updating methods – from recalibration to retraining to model revision. As not all methods will be feasible, permissible, or successful in all situations, research and recommendations are needed to guide updating practice.

#### What updating approaches should be considered?

Although retraining with a cohort of recent observations may be established practice, this approach fails to build on the knowledge encoded in existing models, can be susceptible to overfitting, and may not improve performance above that achieved through recalibration (11, 44–47). For health systems with smaller populations, concerns regarding performance instability when retraining complex models may be more pronounced. Several methods have been developed to compare updating approaches on a particular cohort and recommend the approach that most improves accuracy (17, 45, 46). These methods, however, test for statistically rather than clinically significant differences across potential updates and do not consider whether the recommended update sufficiently restores performance. As methods for establishing clinically relevant decision thresholds mature, testing procedures for selecting updating methods could be implemented with weighted scoring rules to emphasize accuracy in critical regions. Future research should consider expanding options for optimizing decisions using varied performance metrics; increasing test efficiency, particularly for computationally intensive models; methods for evaluating whether updating provides clinically meaningful improvement; and recommendations for cases in which available updating methods do not restore models to acceptable levels of accuracy.

#### How do we handle biased outcome feedback after implementation?

Model updating with current recalibration, retraining, and model revision methods has been developed, evaluated, and applied primarily in research databases. In production systems, interactions between users and AI-enabled decision support tools will, if successful, alter treatment decisions and improve patient outcomes. As a result, the observed data in production systems will be biased and updates using these biased data may reduce future model utility by updating away useful signals (48). These feedback loops created by successful clinical AI tools pose new challenges to updating practice that requires additional methodological research to better characterize the problem; to distinguish between dataset shift and performance changes due to model interventions; and to develop novel algorithms and updating approaches that are robust to confounding by intervention.

## Conclusion

The clinical AI lifecycle is incomplete without components to monitor and stabilize accuracy in evolving clinical environments. Despite the diverse landscape of AI-enabled tools, common challenges to model maintenance impact new and existing implementations regardless of clinical domain and underlying modeling algorithms. Methods development for model monitoring and updating is accelerating, yet open questions for the design of maintenance programs, those described here and more, require additional research and scientific consensus to devise best practices. Establishing best practices is critical to designing AI-enabled tools that deliver reliable predictions, promote adoption, and realize the promise of AI to improve patient care.

## Data Availability

The datasets presented in this article are not readily available because VUMC patient data used in this study are not publicly available. Requests to access the datasets should be directed to Sharon Davis, sharon.e.davis.1@vumc.org.
